# Urinary neutrophil gelatinase-associated lipocalin values alone and combined with Prognostic Index predict septic AKI, DIC, and shock: a pilot study

**DOI:** 10.1186/s13104-020-05232-w

**Published:** 2020-08-18

**Authors:** Yuichiro Shimoyama, Osamu Umegaki, Noriko Kadono, Toshiaki Minami

**Affiliations:** 1 Department of Anesthesiology, Osaka Medical College, Intensive Care Unit, Osaka Medical College Hospital, 2-7 Daigaku-machi, Takatsuki, Osaka 569-8686 Japan; 2 Department of Anesthesiology, Osaka Medical College, Osaka Medical College Hospital, Takatsuki, Japan

**Keywords:** Neutrophil gelatinase-associated lipocalin, Sepsis, Sepsis-3 definition, Inflammation-based prognostic scores, Organ dysfunction

## Abstract

**Objective:**

Sepsis is a syndrome involving life-threatening organ dysfunction. The present study aimed to determine whether septic AKI, ARDS, DIC, and shock can be predicted more readily by combining uNGAL values and inflammation-based prognostic scores, over the use of uNGAL values alone.

**Results:**

ROC curve analyses yielded the following cut-off values: AKI: 438.5 (ng/ml) for uNGAL at Day 1 (AUC, 0.8), 476.9 (ng/ml) for uNGAL at Day 2 (AUC, 0.86), 123.8 (ng/ml) for uNGAL at Day 3 (AUC, 0.81), 133.6 (ng/ml) for uNGAL at Day 4 (AUC, 0.78), 1.0 for iNS NGAL-NLR (AUC, 0.75), 2.0 for iNS NGAL-PI (AUC, 0.77), DIC; 648.5 (ng/ml) for uNGAL at Day 1 (AUC, 0.77); shock; 123.8 (ng/ml) for uNGAL at Day 3 (AUC, 0.71) and 9 for SOFA (AUC, 0.71). Multivariate logistic regression analyses revealed iNS NGAL-PI to be a significant independent predictor of AKI (OR, 20.62; 95% CI, 1.03–412.3; p = 0.048).

## Introduction

In 2016, the Third International Consensus Definitions Task Force (Sepsis-3) proposed new criteria for defining sepsis [1]. Sepsis is a syndrome involving life-threatening organ dysfunction caused by a dysregulated host response to infection. In sepsis patients, an increase in the Sequential Organ Failure Assessment (SOFA) score by ≥ 2 is associated with in-hospital mortality as high as 10% [1].

Neutrophil gelatinase-associated lipocalin (NGAL) is involved in metabolic homeostasis, apoptosis, infection, immune response, and inflammation, and is a potential clinical biomarker for the onset and progression of inflammatory diseases, including arthritic diseases, severe acute pancreatitis, obesity-related metabolic diseases, cardiovascular diseases, AKI, lupus nephritis, intestinal inflammation, and multiple sclerosis [2, 3]. Inflammation-based prognostic scores including the Glasgow Prognostic Score (GPS; calculated based on serum C-reactive protein (CRP) and albumin levels), neutrophil to lymphocyte ratio (NLR), platelet to lymphocyte ratio (PLR), Prognostic Nutritional Index (PNI; calculated based on albumin and lymphocyte counts), and Prognostic Index (PI; calculated based on serum CRP and white blood cell counts) are prognostic biomarkers for several types of cancer [4].

No study to date has assessed the association of septic AKI, ARDS, DIC, and shock with uNGAL values alone or in combination with inflammation-based prognostic scores (i.e., GPS, NLR, PLR, PNI, and PI) in patients with sepsis as defined by the Sepsis-3 definition. Accordingly, the present study aimed to test the following hypotheses: (1) uNGAL values are useful predictors of septic AKI, ARDS, DIC, and shock in sepsis patients, (2) uNGAL values are superior to inflammation-based prognostic scores for predicting septic AKI, ARDS, DIC, and shock, and (3) the ability to predict septic AKI, ARDS, DIC, and shock can be improved by combining uNGAL values and inflammation-based prognostic scores relative to uNGAL values alone.

## Main text

### Methods

#### Patients and study design

This single-center prospective study was conducted in a 16-bed ICU. The study protocol was approved by the Ethics Committee of Osaka Medical College (Osaka, Japan). Adult patients diagnosed with sepsis according to the Sepsis-3 definition [1] and admitted to the ICU were prospectively examined from June 2018 to November 2018. Informed consent was obtained from all patients enrolled in this study or their families. Exclusion criteria were as follows: (1) patients with unstable renal function (increase of sCr by ≥ 0.2 mg/dl in the past month); (2) patients who died within 24 h after being admitted to the ICU; (3) patients on dialysis; (4) patients aged ≤ 18 years, (5) patients who had an immunosuppressive disease (e.g., HIV); and (6) patients undergoing immunosuppressive therapy (e.g., chemotherapy, chronic steroid use, autoimmune disease treatment) within one month of the study.

Urine samples were collected from each patient with a urethral balloon bag to measure urinary NGAL (uNGAL) after ICU entry at the following time points: immediately after and 2, 3, and 4 days after ICU entry. Septic AKI was defined as stage ≥ 1 kidney disease according to the Kidney Disease: Improving Global Outcomes (KDIGO) classification [5]. Septic ARDS was defined according to the Berlin definition [6]. Septic DIC was defined according to the Japanese Association for Acute Medicine DIC diagnostic criteria [7]. Septic shock was defined according to the Sepsis-3 definition [1]. GPS, NLR, PLR, PI, and PNI were examined immediately after ICU entry. For category classification, a total score was calculated (hereafter, “inflammation-NGAL score [iNS]”) as follows: a score of 1 was assigned if uNGAL values and inflammation-based prognostic scores immediately after ICU entry were above cut-offs determined by ROC curve analysis for 28-day mortality; a score of 0 was assigned if the values were below the cut-offs (total score range, 0–2 points). As an example of nomenclature, the combination of uNGAL and NLR is presented as “iNS NGAL-NLR.” uNGAL, inflammation-based prognostic scores, iNS, and changes (Δ) in uNGAL relative to uNGAL values immediately after ICU entry at each sampling point were compared between patients with septic AKI, ARDS, DIC, or shock after ICU entry and those without these disorders.

#### Laboratory assessments

Urine samples were centrifuged at 3500 rpm for 8 min and stored at 4 °C within 12 h after patient enrollment. uNGAL was measured using the ARCHITECT platform (Abbott Japan Co., Ltd., Tokyo, Japan) [8], a chemiluminescent microparticle immunoassay using a noncompetitive, 2-anti-analyte antibody sandwich. In this assay, a microparticle reagent is prepared by covalently attaching an anti-analyte antibody to paramagnetic particles, and a conjugate reagent is prepared by labeling a second anti-analyte antibody with acridinium. The calibrator used for the uNGAL assay was recombinant uNGAL; the highest calibrator sample was 1500 ng/ml uNGAL, with a coefficient of variation of 3.0% at 385 ng/ml [8]. Co-investigators who performed the biomarker assays were blinded to AKI onset.

### Statistical analysis

Categorical data are reported as percentages and compared using Fisher’s exact test. Continuous data are reported as medians with inter-quartile ranges and compared using the Mann–Whitney U test. Receiver operating characteristics (ROC) curves were generated for uNGAL, inflammation-based prognostic scores, iNS, and ΔuNGAL, and areas under the curve (AUCs), cut-off values, sensitivities, and specificities were calculated. With the exceptions of Sequential Organ Failure Assessment (SOFA) and quick SOFA (qSOFA), variables which could be obtained immediately after ICU entry (with P < 0.05 in univariate analysis) were examined further by multivariate logistic regression analysis for septic AKI, ARDS, DIC, and shock. Results of these analyses were then compared with results of multivariate logistic regression analysis performed with SOFA and qSOFA as explanatory variables. P < 0.05 was considered statistically significant. JMP software version 11.0.0 (SAS Institute Inc., NC, USA) was used for all statistical analyses.

## Results

Baseline characteristics of patients are shown in Table 1. No significant differences were observed in age and gender for septic AKI, ARDS, DIC, and shock groups (data not shown).

**Table 1:** Baseline demographic characteristics

Variable	n = 44
Age (years)	74.0 (63.8–78.8)
Gender (male) (%)	29 (65.9)
Cancer (%)	27 (61.4)
Coronary artery disease (%)	1 (2.3)
Diabetes mellitus (%)	4 (9.1)
Hypertension (%)	10 (25.0)
Albumin (g/dL)	2.3 (1.8–3.1)
CRP (mg/dL)	9.3 (2.5–19.0)
WBC (× 10^9^ l^−1^)	11.8 (5.7–17.8)
Neutrophil count (× 10^9^ l^−1^)	8.8 (2.9–15.7)
Lymphocyte count (× 10^9^ l^−1^)	0.5 (0.2–0.7)
Platelet count (× 10^4^ mm^−3^)	17.8 (11.4–25.3)
Fibrinogen (mg/dL)	537 (318–730)
Survival (deceased) (%)	12 (27.3)
Septic AKI (%)	20 (45.5)
Septic ARDS (%)	5 (11.4)
Septic shock (%)	27 (61.4)
Septic DIC (%)	15 (34.1)
NGAL: Day 1 (ng/mL)	247.9 (77.1–634.6)
NGAL: Day 2 (ng/mL)	268.9 (127.9–1098.9)
NGAL: Day 3 (ng/mL)	123.8 (63.1–461.7)
NGAL: Day 4 (ng/mL)	120.7 (50.2–638.1)
ΔNGAL: Day 2-Day 1 (ng/mL)	0.5 (−140.6–129.4)
ΔNGAL: Day 3-Day 1 (ng/mL)	−10.6 (−198.2–90.7)
ΔNGAL: Day 4-Day 1 (ng/mL)	−14.75 (−312.4–86.3)
GPS	1 (1–2)
NLR	17.7 (5.3–37.7)
PLR	373.0 (195.7–540.9)
PI	1 (0–2)
PNI	26.5 (20.2–33.6)
SOFA	9 (6–11)
qSOFA	2 (1–3)

*CRP* C-reactive protein, *WBC* white blood cell, *AKI* acute kidney injury, *ARDS* acute respiratory distress syndrome, *DIC* disseminated intravascular coagulation, *NGAL* neutrophil gelatinase-associated lipocalin, *GPS* Glasgow prognostic score, *NLR* neutrophil to lymphocyte ratio, *PLR* platelet to lymphocyte ratio, *PI* prognostic index, *PNI* prognostic nutritional index, *qSOFA* quick Sequential Organ Failure Assessment

ROC curve analyses (Table 2) yielded the following cut-off values: AKI: 438.5 (ng/ml) for uNGAL at Day 1 (AUC, 0.8; sensitivity, 65%; specificity, 83%), 476.9 (ng/ml) for uNGAL at Day 2 (AUC, 0.86; sensitivity, 71%; specificity, 91%), 123.8 (ng/ml) for uNGAL at Day 3 (AUC, 0.81; sensitivity, 92%; specificity, 74%), 133.6 (ng/ml) for uNGAL at Day 4 (AUC, 0.78; sensitivity, 67%; specificity, 71%), 1.0 for iNS NGAL-NLR (AUC, 0.75; sensitivity, 75%; specificity, 75%), 2.0 for iNS NGAL-PI (AUC, 0.77; sensitivity, 50%; specificity, 92%),DIC; 648.5 (ng/ml) for uNGAL at Day 1 (AUC, 0.77; sensitivity, 60%; specificity, 97%); shock; 123.8 (ng/ml) for uNGAL at Day 3 (AUC, 0.71; sensitivity, 68.4%; specificity, 75%) and 9 for SOFA (AUC, 0.71; sensitivity, 73.1%; specificity, 64.7%).

**Table 2:** Receiver operating curve analysis

Variable	AUC	Cut-off	*P* value	Sensitivity (%)	Specificity (%)
Septic AKI					
NGAL (ng/mL) Day 1	0.80	438.50	< 0.001	65.0	83.3
NGAL (ng/mL) Day 2	0.86	476.90	< 0.001	70.6	90.5
NGAL (ng/mL) Day 3	0.81	123.80	< 0.001	91.7	73.7
NGAL (ng/mL) Day 4	0.78	133.60	0.002	66.7	70.6
iNS NGAL-NLR	0.75	1.00	< 0.001	75.0	75.0
iNS NGAL-PI	0.77	2.00	< 0.001	50.0	91.7
Septic DIC					
NGAL (ng/mL) Day 1	0.77	648.50	0.001	60.0	96.6
Septic shock					
NGAL (ng/mL) Day 3	0.71	123.80	0.031	68.4	75.0
SOFA	0.71	9.00	0.009	73.1	64.7

*AUC* area under the curve, *AKI* acute kidney injury, *NGAL* neutrophil gelatinase-associated lipocalin, *iNS* inflammation-NGAL score, *NLR* neutrophil to lymphocyte ratio, *PI* prognostic index, *DIC* disseminated intravascular coagulation, *SOFA* Sequential Organ Failure Assessment

Data on variables which could be obtained immediately after ICU entry and were significant (P < 0.05) in univariate analysis (Table 3) were examined further by multivariate logistic regression analysis for septic AKI, ARDS, DIC, and shock. The analyses revealed iNS NGAL-PI to be a significant independent predictor of AKI (OR, 20.62; 95% CI, 1.03–412.3; p = 0.048; Additional file 1: Table S1). On the other hand, uNGAL alone was not identified as a significant independent predictor by multivariate logistic regression analysis.

**Table 3:** Predictive factors for septic AKI, ARDS, Shock, and DIC (univariate analysis)

Variable	AKI (n = 20)	ARDS(n = 5)	Shock (n = 27)	DIC (n = 15)
	*P* value	*P* value	*P* value	*P* value
Age	0.822	0.335	0.708	0.264
Gender	0.246	0.480	1.000	1.000
Cancer	0.651	0.359	0.526	0.333
Coronary artery disease	0.356	1.000	0.386	1.000
Diabetes mellitus	0.213	0.394	0.634	1.000
Hypertension	0.036	0.586	0.158	1.000
Albumin	0.723	0.810	0.141	0.275
CRP	0.038	0.671	0.588	0.116
WBC	0.120	0.365	0.152	0.328
Neutrophil	0.120	0.385	0.233	0.665
Lymphocytes	0.706	0.839	0.057	0.012
Platelet count	0.540	0.739	0.177	0.000
Fibrinogen	0.448	0.825	0.043	0.072
Survival	0.103	0.116	0.090	0.284
Septic AKI		0.160	0.124	0.059
Septic ARDS	0.099		0.634	1.000
Septic shock	0.090	0.634		0.021
Septic DIC	0.042	1.000	0.021	
NGAL: Day 1	0.001	0.189	0.049	0.003
NGAL: Day 2	0.000	0.401	0.120	0.054
NGAL: Day 3	0.004	0.547	0.057	0.139
NGAL: Day 4	0.019	0.773	0.096	0.157
ΔNGAL: Day 2-Day 1	0.736	0.267	0.794	0.332
ΔNGAL: Day 3-Day 1	0.351	0.423	0.417	0.459
ΔNGAL: Day 4-Day 1	0.571	0.962	0.824	0.707
GPS	0.390	0.709	0.935	0.222
NLR	0.300	0.471	0.990	0.480
PLR	0.572	0.753	0.691	0.304
PI	0.084	0.596	0.299	0.885
PNI	0.860	0.725	0.101	0.113
iNS- GPS	0.024	0.158	0.688	0.045
iNS- NLR	0.001	0.756	0.115	0.021
iNS- PLR	0.022	0.472	0.284	0.492
iNS- PI	0.001	0.264	0.308	0.064
iNS- PNI	0.043	0.419	0.840	0.273
SOFA	0.723	0.568	0.020	0.348
qSOFA	0.294	0.493	0.309	0.508

*AKI* acute kidney injury, *ARDS* acute respiratory distress syndrome, *DIC* disseminated intravascular coagulation, *CRP* C-reactive protein, *WBC* white blood cell, *NGAL* neutrophil gelatinase-associated lipocalin, *GPS* Glasgow Prognostic Score, *NLR* neutrophil to lymphocyte ratio, *PLR* platelet to lymphocyte ratio, *PI* prognostic index, *PNI* prognostic nutritional index, *iNS* inflammation-NGAL score, *qSOFA* quick Sequential Organ Failure Assessment

## Discussion

ROC curve analyses identified the following variables to be predictors of septic AKI, DIC, and shock: uNGAL at Days 1–4, iNS NGAL-NLR, and iNS-PI for septic AKI; uNGAL Day 1 for septic DIC; and uNGAL Day 3 and SOFA for septic shock (Table 2). Multivariate analysis revealed iNS NGAL-PI to be a predictor for septic AKI (Additional file 1: Table S1). On the other hand, inflammation-based prognostic scores, when used alone, did not serve as significant prognostic predictors based on AUC, sensitivity, and specificity determined by ROC curve analysis and univariate analysis (data not shown).

Sepsis involves the activation of both pro- and anti-inflammatory responses [9], with modifications in non-immunologic pathways including cardiovascular, neuronal, autonomic, hormonal, bioenergetic, metabolic, and coagulation pathways [10–12]]. NGAL is associated with a variety of physiological and pathophysiological processes including metabolic homeostasis, apoptosis, infection, immune response, and inflammation [3]. Vanmassenhove et al. reported that serum NGAL levels increase in parallel with the severity of sepsis, illness, and inflammation in sepsis patients [13]. The present study found that uNGAL can serve as a biomarker for various complications related to sepsis, including not only septic AKI, but also septic DIC and shock. This likely involves the above-mentioned characteristics of NGAL.

The AUC, sensitivity, and specificity of iNS NGAL-PI for predicting AKI were 0.769, 50, and 92%, respectively, corresponding to a higher specificity than those observed for uNGAL Days 1–4 alone. These findings suggest that combining PI with uNGAL values may serve as an easy “rule in” test. The AUC, sensitivity, and specificity of iNS NGAL-PI were also higher than those of SOFA and qSOFA (data not shown). Multivariate analyses revealed that higher iNS NGAL-PI values corresponded to a stronger ability to predict AKI (OR, 20.62; p = 0.048) (Table S1), and that iNS NGAL-PI more accurately predicted AKI than uNGAL alone, SOFA, and qSOFA in sepsis patients. The advantage of combining biomarkers to improve their performance for predicting AKI has been reported in previous studies [14–16]. In those studies, urinary biomarkers that reflect renal tubular injury such as NGAL, kidney injury molecule-1, L-type fatty acid-binding protein, and N-acetyl-β-D-glucosaminidase were combined to predict post-cardiac surgery AKI. As mention above, sepsis and septic AKI are highly complex and involve multiple pathophysiological mechanisms [17]. For such complex conditions, combinations of biomarkers involved in different pathways may help make accurate predictions at an early stage.

We previously reported that NLR is superior to other inflammation-based prognostic scores in predicting the mortality of patients with gastrointestinal perforation and pneumonia [18, 19]. In the context of AKI, however, the present study found PI to be a predictor of septic AKI. PI reportedly predicts the prognosis of patients with lung cancer [20]]. According to a study by Proctor et al., which involved comparing the prognostic value of numerous inflammation-based prognostic scores (modified GPS, NLR, PLR, PI, and PNI) for various cancers, modified GPS and PI were found to have prognostic value for cancer independent of the tumor site [21]. Our findings suggest that, even among the various variables which can be tested to assess inflammation, CRP and lymphocytes (which are used to calculate PI) are important for diagnosing septic AKI.

No previous study has assessed the association between uNGAL alone or uNGAL combined with PI and septic AKI, DIC, and shock in patients with sepsis defined according to the Sepsis-3 definition. Our findings are novel in this respect. Further studies will be needed to clarify the mechanism underlying the predictive value of uNGAL and PI for septic AKI.

### Conclusion

In conclusion, uNGAL and iNS NGAL-PI were found to be predictors of septic AKI, DIC, and shock. Combining PI with uNGAL values improved the predictive value of uNGAL for septic AKI. Moreover, iNS NGAL-PI was found to more strongly predict septic AKI in sepsis patients than SOFA and qSOFA. Further studies aimed at understanding the exact role of uNGAL and PI in predicting septic AKI, DIC, and shock are warranted.

## Limitation

This study has several limitations. First, this was a single-center study with a small cohort. Second, this study used a single biomarker, and no comparisons were made with other biomarkers.

## Supplementary information

**Additional file 1: Table S1.** Predictive Factors for Septic AKI, ARDS, Shock, and DIC (Multivariate Analysis).

## Data Availability

The datasets used and/or analyzed during the current study are available from the corresponding author on reasonable request.

## Abbreviations

SOFA: The Sequential Organ Failure Assessment
NGAL: Neutrophil gelatinase-associated lipocalin
GPS: Glasgow Prognostic Score
CRP: C-reactive protein (CRP)
NLR: Neutrophil to lymphocyte ratio
PLR: Platelet to lymphocyte ratio
PNI: Prognostic Nutritional Index
PI: Prognostic Index
ROC: Receiver operating characteristics
AUC: Areas under the curve
iNS: Inflammation-NGAL score
